# Whole-Exome Sequencing Improves Understanding of Inherited Retinal Dystrophies in Korean Patients

**DOI:** 10.3390/cimb46100654

**Published:** 2024-09-29

**Authors:** Youngchan Park, Youngjin Kim, Insong Koh, Jong-Young Lee

**Affiliations:** 1 Department of Biomedical Informatics, Hanyang University, Seoul 04763, Republic of Korea; ycpark1228@korea.kr; 2 Division of Bio Bigdata, Department of Precision Medicine, Korea National Institution of Health, KCDC, Cheongju 28159, Republic of Korea; 3 Elite Eye Hospital, Seoul 03779, Republic of Korea; yjkim@eliteeye.co.kr; 4 OneOmics Co., Ltd., Gimpo-si 14585, Republic of Korea

**Keywords:** retinitis pigmentosa (RP), Korean families, whole-exome sequencing (WES), novel variants

## Abstract

Retinitis pigmentosa (RP) encompasses a diverse range of hereditary, degenerative retinal ailments, presenting notable obstacles to molecular genetic diagnoses due to the intricate array of variants in different genes involved. This study enrolled 21 probands and their families who have been diagnosed with nonsyndromic RP but without a previous molecular diagnosis. We employed whole-exome sequencing (WES) to detect possible harmful gene variations in individuals with unknown-cause RP at the molecular level. WES allowed the identification of ten potential disease-causing variants in eight different genes. In 8 out of the total 21 patients, this method successfully identified the underlying molecular causes, such as putative pathogenic variants in genes including *CRB1*, *KLHL7*, *PDE6B*, *RDH12*, *RP1*, *RPE65*, *USH2A*, and *RHO*. A novel variant was identified in one of these genes, specifically *PDE6B*, providing valuable information on prospective targets for future enhanced gene therapeutic approaches.

## 1. Introduction

Approximately one in every four thousand individuals worldwide is afflicted with RP, the most common form of inherited retinal dystrophies (IRDs). RP (MIM: 268,000) is defined by the primary degeneration of rods, often presenting as progressive night blindness and subsequent visual field constriction. The condition begins with cones malfunctioning, leading to decreased visual acuity and central vision loss [1,2]. Moreover, in most patients, a symptom known as “bone spicule” pigmentation is observed in the retina. It is marked by the gradual degeneration of rod and cone photoreceptors, resulting in significant vision impairment in both eyes [3]. Cmmon symptoms encompass advancing nyctalopia, visual field constriction, and visual acuity decrease.

Usually, RP patients share similar genetic backgrounds, with phenotypic heterogeneity among people. Furthermore, RP is a genetically diverse illness with autosomal-dominant, autosomal-recessive, or X-linked inheritance. Genetic, allelic, and phenotypic variation make the diagnosis of RP patients a difficult process. About 93 genes are linked to nonsyndromic RP variants, and the RetNet database (https://web.sph.uth.edu/RetNet/ (accessed on 20 May 2023)) provides up-to-date information [4,5].

This research aimed to discover the putative pathogenic gene associated with Korean families exhibiting retinitis pigmentosa via whole-exome sequencing. Furthermore, we aimed to assess the diagnostic efficacy of this approach and to determine the relationship between candidate genes and clinical characteristics.

## 2. Materials and Methods

### 2.1. Clinical Examination

A group of 21 families who were diagnosed with RP but did not have any previous molecular test results, and who had a well-documented family history, were chosen from an elite eye clinic in Seoul, Korea, between January 2021 and October 2022.

All patients visiting the elite eye clinic received comprehensive ophthalmic examinations, including best-corrected visual acuity (BCVA) and intraocular pressure (IOP) measurements, slit lamp biomicroscopy, color fundus photography (Topcon Inc., Tokyo, Japan), ocular biometry applying optical low-coherence reflectometry (Lenstar 900 Optical Biometer, Haag-Streit, Koeniz, Switzerland), OCT and OCT angiography (Heidelberg Engineering, Max-Jarecki-Straße 8, 69115 Heidelberg, Germany), and stationary perimetry tests (Humphery field analyzer; Carl Zeiss Meditec, Inc., Dublin, CA, USA).

Written informed consent was obtained from all participants or their guardians, and the study received approval from a Bioethics Committee authorized by the Ministry of Health and Welfare (MOHW) of and Hanyang University and OneOmics. All study protocols followed the principles of the Declaration of Helsinki.

### 2.2. Whole-Exome Sequencing

Using Exgene Blood SV mini (GeneAll, Seoul, Republic of Korea), DNA was extracted from the blood or saliva of patients and their families according to the manufacturer’s protocol [6]. Exomes were captured and amplified via PCR by using a xGen Exome Research Panel V2 (Integrated DNA Technologies, Coralville, IA, USA) exome kit [7]. Paired-end sequencing was performed using Novaseq 6000 (Illumina, San Diego, CA, USA) [8]. Variant discoveries were performed using TAK’s best practice. Discovered variants were functionally annotated by utilizing the software tool snpEff (version 5.0) in conjunction with the genome annotation release GRCh38.102 [9]. To assess in silico variant effects, dbNSFP v4.5 (Gencode release 29/Ensembl version 94) was annotated using SnpSift v5.0 [10,11]. dbSNP build 155 and the CLINVAR database were annotated using bcftools v1.3 [12,13,14]. Variants have a frequency of more than 1% in GnomAD and the KRGDB [15,16,17].

To avoid missing disease-related variations with an allele frequency of 1% or higher, the detected variants were filtered based on an allele frequency of below 1% in the KRGDB [18]. Each variant that successfully passed the screening process was examined against 93 genes associated with RP as listed in the RetNet database [19].

The pathogenicity of the chosen variants was assessed based on the norms and recommendations set out by the American College of Medical Genetics and Genomics (ACMG) guidelines using Intervar [20,21]. GATK-gCNV analysis allows for a germline copy number variation (CNV) pipeline [22].

### 2.3. Sanger Confirmation

The novel candidate variations were confirmed via capillary sequencing. Sanger sequencing was conducted on the relevant gene segments to conduct a segregation analysis and determine the inheritance pattern of the modified alleles in specific families using the DNA samples of available relatives.

## 3. Results

The present study includes 21 patients who have been diagnosed with nonsyndromic RP. The average age at which RP was diagnosed was 31.72 ± 12.76 years (mean ± SD), whereas the mean age of examination was assessed as being 37.78 ± 12.39 years (Table 1). All participants had typical characteristics of RP, such as the first manifestation of night blindness being the primary symptom, followed by a gradual reduction in the visual field over subsequent years.

**Figure 1:**
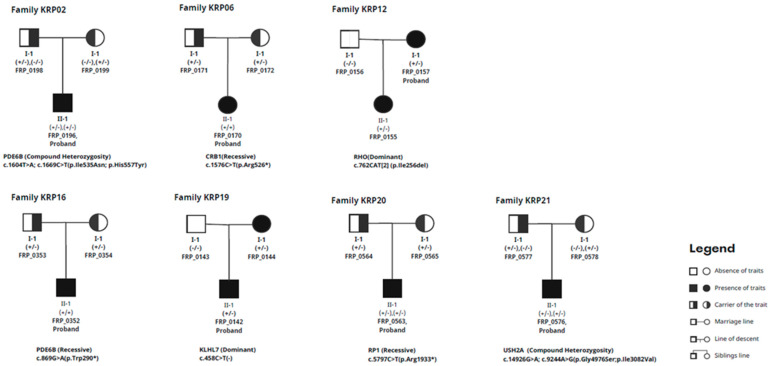
Full pedigree chart of trio families.

Cataracts were detected in patients FRP_0196, FRP_0170, FRP_0043, FRP_0207, FRP_0355, FRP_0157, FRP_0221, and FRP_0243. Detailed ophthalmological observations of the patients are presented in Table 1, and the clinical characteristics of family with novel variants of *PDE6B* gene are documented in Figure 1.

Thirteen families had cases that were not resolved even after WES was performed. WES identified potentially causal variations in genes associated with RP in 8 out of the 21 families (Table 2). These variants include one new variant and eight variants that have been previously described. The genes involved include *PDE6B*, *CRB1*, *PRE65*, *RHO*, *KLHL7*, *RP1*, *and USH2A*. The in silico investigation of CNVs utilizing GATK’s gCNV did not detect any noteworthy structural changes. In addition, variants from the remaining 13 families did not meet the criteria outlined by the ACMG standard.

**Figure 2:**
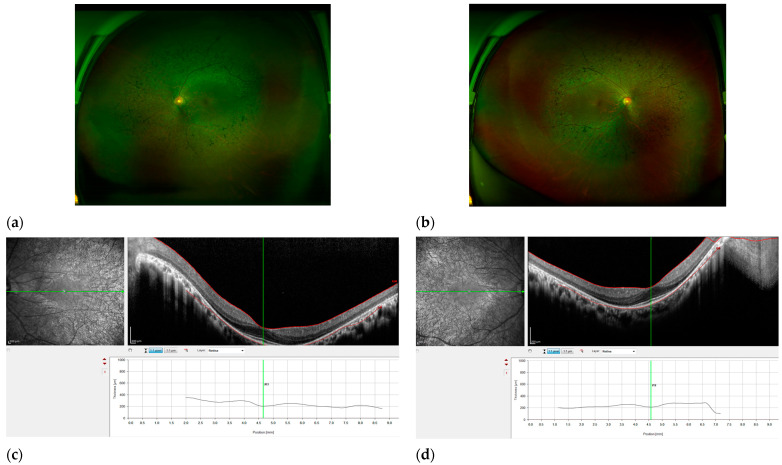
Features of RP patient KRP16. Fundus photography of the right eye (**a**) and left eye (**b**), exhibiting characteristic traits of RP, such as optic disc pallor, narrowed retinal arteries, and pigmentation resembling bone spicules in the mid-peripheral area. Scale bar, 200 μm. (**c**,**d**) OCT revealed a reduction in the thickness of the retina and the absence of photoreceptor cells outside the fovea. The right eye has observable solitary cystic alterations in the foveola, as well as a delicate epiretinal membrane in the temporal region of the macula (scale bar, 200 μm).

A novel variant was identified from gene *PDE6B* (c.869G>A, p.Trp290*). On *PDE6B*, c.869G>A(p.Trp290*) is a putative pathogenic novel variant in the KRP16 family. The proband was diagnosed with retinitis pigmentosa (Figure 2). The patient did not exhibit any indications or symptoms that were present in his mother and brother. The variant tester agreed on the potential impact of stop-gain (automatically causing disease).

## 4. Discussion

Inherited RP impacts people of all age groups and covers a wide range of diseases with significant genetic and phenotypic diversity. The pathogenesis of these disorders involves pathogenic variations that may vary from single-nucleotide changes to chromosomal rearrangements [23].

These variants impact genes that encode various signaling and structural components. Several genetic investigations have been carried out to comprehend the hereditary foundations of RP, encompassing molecular genetic research and studies on the aggregation of families. These investigations have revealed a genetic predisposition causing RP, revealing many pathogenic genes or susceptibility regions that are significantly linked to its development in Korean population specifically.

Trio-based WES is a more comprehensive method compared to WES focused on a single individual (proband-centered WES). This approach allows for the discovery of gene variations and a thorough evaluation of their pathogenicity. Additionally, it permits the investigation of gene variants using the principles of cogenetic segregation. This methodology can create an improved strong network that maps the association between genes and phenotypes, enabling a more comprehensive exploration of the inheritance patterns of mutant genes. In addition, because of the scarcity of genetic studies focusing on IRDs and the urgent need for RP research, specifically within the unique population of Korea, we recruited a group of 21 families who have been diagnosed with RP. Using a trio-based WES technique, our goal is to obtain a more comprehensive understanding of the genetic factors involved in RP. Specifically, we seek to uncover how genetic variations contribute to the development of this disorder within families.

The variant p.Trp290* is believed to cause the denaturation of PDE6B by interfering with the structure of the GAF2 region of the protein [24]. Phosphodiesterase 6B (PDE6B) variants often cause autosomal recessive retinitis pigmentosa, also known as rod–cone dystrophy. The PDE6B protein plays a crucial role in phototransduction. It is crucial to comprehend the pathogenicity and functional significance of identified PDE6B polymorphisms to provide genetic information to families and facilitate participation in therapeutic trials for autosomal-recessive PDE6B-related retinitis pigmentosa. PDE6A and PDE6B combine to create a heterodimer that is blocked by a homodimer consisting of two γ subunits, which are produced by PDE6G. PDE6A and PDE6B each contain two potential noncatalytic domains (GAF1 and GAF2) in the N-terminus, which interact with cGMP and the polycationic region of two γ subunits in the inhibitory state of the complex [25].

Given that the one cause of RP materializes through genetic influence, our study includes an examination of documented variants in RP risk genes: PDE6B, CRB1, RPE65, RHO, KLHL7, RP1, and USH2A. These genes hold pivotal roles in bone spicule formation, modulating scleral thickness, influencing choroidal blood flow, and orchestrating other factors germane to the onset of RP. The identification of pathogenic variants in these genes promises to enrich our understanding of the pathogenesis of RP.

The objective of this work was to conduct a multimodal study that could characterize a unique form of IRD in Korean population, broadening understanding of how a novel pathogenic variant affects the phenotype differently from a similar one [26].

To gain a more comprehensive understanding of the genetic components that contribute to RP, it is essential to have a broader sequencing coverage that encompasses noncoding areas. This may be achieved by utilizing advanced technologies like WGS, along with a larger collection of well-organized clinical data. Moreover, it is crucial to carry out additional functional investigations and ex vivo experimental validations to verify the several risk genes for RP that have been found in our research.

To summarize, our study’s thorough investigation of RP families from Korea tackles a gap in genetic research in this area. Through the consolidation and integration of data, our goal is to create a helpful resource for genetic counselling and personalized treatment strategies.

## Figures and Tables

**Table 1:** Clinical symptoms and signs in RP patients. BCVA: best-corrected visual acuity; IOP: intraocular pressure; OD: right eye; OS: left eye; SPH: sphere (the correction for nearsightedness or farsightedness is spherical, equal in all meridians of the eye); LP: light perception; HM: hand motion; f/c: finger count; PL: plano (no nearsightedness or farsightedness correction); RNFLD: retinal nerve fiber layer defect; and ERM: epiretinal membrane.

Family ID	Patient ID	Onset Age	Age	Anterior Segmentation	Diagnosis		BCVA		IOP	
					OPTOS	OCT	OD	OS	OD	OS
KRP01	FRP_0106	32	35	-	OD: bone spicule OS: retinal degeneration	OD: epiretinal membrane OU: photoreceptor defect	0.4	0.4	20	20
KRP02	FRP_0196	30	36	Cataract	OU: multiple pigmentosa, waxy disc, and macular mottling	OU: RNFLD, ERM OD: macular cyst	0.5	0.7	15	15
KRP03	FRP_0036	22	22	-	OU: no hemorrhage and exudate, bone spicule	OU: RNFLD (with normal limits)	1	1	15	16
KRP04	FRP_0004	32	42	-	OU: pale waxy disc, bone spicule	OU: RNLFD, thin retina and cell destruction	HM	LP(+)	13	13
KRP05	FRP_0124	42	45	-	OU: pale waxy disc, bone spicule	OU: RNLFD, photoreceptor defect	0.2	0.3	14	15
KRP06	FRP_0170	28	29	Cataract	LCA (Leber congenital amaurosis) No eyesight from 14 yr		LP(+)	LP(+)	8	10
KRP07	FRP_0043	36	50	Cataract	OU: pale waxy disc, bone spicule	OU: RNLFD, photoreceptor defect	0.4	0.4	18	19
	FRP_0046	46	55	-	OU: pale waxy disc, bone spicule	OU: RNFLD OS: ERM with macular edema	0.4	0.4	18	19
KRP08	FRP_0207	53	60	Cataract	OU: multiple pigmentation	OU: RNFLD, photoreceptor destruction, and multiple pigmentation	HM	LP(+)	17	17
KRP09	FRP_0355	18	32	Cataract	OU: multiple pigmentation	OU: multiple pigmentation	0.9	0.8	17	16
KRP10	FRP_0150	27	28	-	OU: multiple bone spicules	OU: photoreceptor defect	f/c 50 cm	f/c 50 cm	16	14
KRP11	FRP_0342	19	19	-	OU: multiple pigmentation	OU: RNFLD (with normal limits)	1	1	11	10
KRP12	FRP_0155	24	27	-	OU: pale waxy disc, multiple bone specular pigmentation	OU: RNFLD, multiple pigmentation	0.4	0.3	21	21
	FRP_0157	64	65	Cataract	OU: pale waxy disc, multiple bone specular pigmentation	OU: RNFLD, multiple pigmentation OS: ERM	HM	HM	18	18
KRP13	FRP_0191	21	26	-	OU: pale waxy disc, multiple bone specular pigmentation	OU: multiple pigmentation	0.7	1	13	12
KRP14	FRP_0221	32	38	Cataract	OS: retinal hemorrhage OU: retinal pigmentation	OU: RNFLD OS: macular edema, retinal hemorrhage	f/c 20 cm	1	14	15
KRP15	FRP_0243	48	48	Cataract	OU: multiple pigmentation	OU: RNFLD, photoreceptor defect	0.1	0.05	12	10
KRP16	FRP_0352	21	34	-	OU: multiple pigmentation	OU: RNFLD, photoreceptor partial destruction	1	1	14	15
KRP17	FRP_0546	10	19	-	OU: multiple pigmentation	OU: RNFLD, photoreceptor defect	f/c 50 cm	0.5	13	15
KRP18	FRP_0434	-	44	-	OU: multiple pigmentation, pale waxy disc	OU: RNFLD, photoreceptor defect	HM	0.9	17	15
KRP19	FRP_0142	32	33	-	OU: bone spicule	OD: ERM OU: photoreceptor defect	LP(+)	LP(+)	10	9
KRP20	FRP_0563	24	40	-	OU: macular mottling	OU: macular degeneration	0.6	0.6	15	16
KRP21	FRP_0576	37	42	-	OU: multiple pigmentation	OU: multiple pigmentation	0.7	0.7	13	12

**Table 2:** Pathogenic DNA variants in RP patients. Variants of the *PED6B gene (KRP16 family) have not been reported previously.

Fam ID	Gene	Heredity	RSID	Variant	Protein Changes	Variant Tester	PROVEAN	Polyphen2	SIFT	REVEL Score	gNOMAD Freq.(4.1.0)	ACMG Criteria	ClinVar Pathogenicity
KRP02	*PDE6B*	Recessive (CH)	rs527236088	c.1604T>A	p.Ile535Asn	Disease-causing	Damaging	Probably damaging	Damaging	0.818	0.000006158	Pathogenic, Pathogenic	Pathogenic/likely pathogenic
		Recessive (CH)	rs121918581	c.1669C>T	p.His557Tyr	Disease-causing (automatic curation)	Damaging	Probably damaging	Damaging	0.991	0.00001539	Pathogenic, Pathogenic	Pathogenic/likely pathogenic
KRP06	*CRB1*	Recessive	rs114342808	c.1576C>T	p.Arg526*	Disease-causing (automatic curation)	NA	NA	NA	NA	0.00001163	Pathogenic	Pathogenic
KRP12	*RHO*	Dominant	rs1553781360	c.762CAT [2]	p.Ile256del	NA	NA	NA	NA	NA	NA	NA	Conflicting interpretations of pathogenicity
KRP16	*PDE6B*	Recessive	rs1241365850	c.869G>A	p.Trp290*	Disease-causing (automatic curation)	NA	NA	NA	NA	NA	NA	NA
KRP19	*KLHL7*	Dominant	rs137853113	c.458C>T	NA	Disease-causing (automatic curation)	Damaging	Probably damaging	Damaging	0.895	NA	Pathogenic (PS2 + PM1 + PM2 + PP3)	Pathogenic
KRP20	*RP1*	Recessive	rs118031911	c.5797C>T	p.Arg1933*	Disease-causing	NA	NA	NA	NA	0.0001478	Pathogenic	Conflicting interpretations of pathogenicity
KRP21	*USH2A*	Recessive (CH)	rs200761611	c.14926G>A	p.Gly4976Ser	Disease-causing	Damaging	Probably damaging	Damaging	0.372	0.00001642	Uncertain significance	Conflicting interpretations of pathogenicity
		Recessive (CH)	rs527689947	c.9244A>G	p.Ile3082Val	Polymorphism	Neutral	Benign	Tolerate	0.486	0.00003901	Likely pathogenic (PS2 + PM2)	Variants of uncertain significance

## Data Availability

The data used in this study are available from the corresponding author upon request.
